# 

**Published:** 1892-08

**Authors:** 


CONTENTS.

VOL. 39. No. 8.

| | PAGE. |
|---|---|
| Talks with Dr. Mandeville...... | 169 |
| The Liver...................... | 172 |
| Real, Idealistic or Materialistic | 175 |
| Russian Lepers .......... ..... | 178 |
| The Wonders of your Watch... | 180 |
| A Spirit Whisper .............. | 181 |
| A True View of Marriage...... . | 183 |
| Overwork and Disease .......... | 183 |
| How Much They Know ........ | 184 |
| Tired People. ............... | 185 |
| The Proper Way to Sit.... ..... | 186 |
| Glycerine in the House........ | 187 |
| Women and the Marriage Laws | 187 |
| Cooking and Digestion ...... . | 187 |
| Hints for the Bath............. | 188 |
| Miscellaneous .... .......... | 188 |
| Literary.......... ............ | 191 |
| My Creed........ .............. | 192 |

INDEX TO ADVERTISEMENTS OF HALF A PAGE AND OVER.

| | PAGE. |
|---|---|
| Christian Life.... .......... | I |
| Magic Fuel Co. ........ ..... | II |
| Bovinine..................... | IV |
| Ayer's Cathartic Pills........ | V |
| Hall's Journal Premiums. ... | VII |
| Washington Improvement Co. | VIII |
| Herba Vita..... ......... . | X |
| Royal Baking Powder....... | XI |
| Buffalo Lithia Water...... . | XI |

